# Focusing on Mouth Movement to Improve Genuine Smile Recognition

**DOI:** 10.3389/fpsyg.2020.01126

**Published:** 2020-07-28

**Authors:** Qian-Nan Ruan, Jing Liang, Jin-Yu Hong, Wen-Jing Yan

**Affiliations:** ^1^Wenzhou 7th People’s Hospital, Wenzhou, China; ^2^School of Educational Science, Ludong University, Yantai, China; ^3^College of Education, Wenzhou University, Wenzhou, China

**Keywords:** genuine and posed smiles, Duchenne marker, mouth movement, perceptual-attentional hypothesis, dynamic lip, training

## Abstract

Smiles are the most commonly and frequently used facial expressions by human beings. Some scholars claimed that the low accuracy in recognizing genuine smiles is explained by the perceptual-attentional hypothesis, meaning that observers either did not pay attention to responsible cues or were unable to recognize these cues (usually the Duchenne marker or AU6 displaying as contraction of muscles in eye regions). We investigated whether training (instructing participants to pay attention either to the Duchenne mark or to mouth movement) might help improve the recognition of genuine smiles, including accuracy and confidence. Results indicated that attention to mouth movement improves these people’s ability to distinguish between genuine and posed smiles, with nullification of the alternative explanations such as sample distribution and intensity of lip pulling (AU12). The generalization of the conclusion requires further investigations. This study further argues that the perceptual-attentional hypothesis can explain smile genuineness recognition.

## Introduction

Facial expressions are the primary channel used by humans to express social intent. Among the various human facial expressions, smiles are the most common and frequent. Smiles are often expressed during social interactions, representing a powerful signal of affiliative behavior, cooperation, and social bonding (Tomkins, 1962; Bachorowski and Owren, 2001; Martin et al., 2017). Smiling individuals are perceived as happier (Otta et al., 1996), more attractive, communal, competent (Matsumoto and Kudoh, 1993; Hess et al., 2002), likable (Palmer and Simmons, 1995), approachable, friendly, and honest (Centorrino et al., 2015). A smile from another promises a safe and satisfying interaction (Krys et al., 2016). That is why people tend to produce smiles frequently and voluntarily. Smiles, however, can easily be faked (Mehu, 2011). Consequently, the perceiver has a vested interested in examining their spontaneity.

However, the ability to accurately distinguish between genuine and posed smiles is far from common. Some studies have reported that generally the level of accuracy is around 55% (Frank et al., 1993; Gosselin et al., 2002b); others have argued that it is closer to 70% (Boraston et al., 2008; Manera et al., 2011), with large individual differences. Moreover, when participants are asked to identify whether two types of smiles are the same, the “same group” tends to be much larger than the “different group” (Perron and Roy-Charland, 2013). The present research studied the differences between genuine and posed smiles and trained people to improve their recognition of smile spontaneity.

Ekman and Friesen (1982); Frank and Ekman (1993), and Frank et al. (1993) drew distinctions and specified major differences between felt emotional smiles (i.e., genuine expressions) and false smiles deliberately shown to simulate enjoyment (i.e., posed expressions). One of the most replicated and best-documented criteria for this differentiation (Frank and Ekman, 1993) is the Duchenne smile, which consists of AU6 and AU12 (displaying as pull-up lip corners) and can be used as an indicator for distinguishing genuine from posed smiles. According to the Facial Action Coding System (Ekman et al., 2002), which delineates dozens of relatively independent action units (AUs) based on the anatomical characteristics of human facial muscles, AU6 indicates the contraction of the orbicularis oculi, which is usually expressed as crow’s feet; AU12 indicates a contraction of the zygomaticus major, which is manifested by the extension of the mouth to the sides and upward. Only when the two AUs appear at the same time (AU6 and AU12) is the smile considered genuine (Ekman, 2003; Krumhuber and Manstead, 2009). Most previous research has focused on this morphological smile marker and its purported link to positive emotions (see Kappas and Descôteaux, 2003). According to Frank and Ekman (1993), most people can control AU12 autonomously, while only a few (i.e., 20%) can autonomously control AU6. A meta-analysis confirmed this conclusion that people producing Duchenne smiles are rated more positively than those displaying non-Duchenne smiles (Gunnery and Ruben, 2016).

Yet there is a significant controversy regarding whether AU6 can be used as a criterion for distinguishing between genuine and posed smiles, because some people can display Duchenne smiles on their own or when in an unpleasant mood. Krumhuber and Manstead (2009) compared smiles under genuine and enacted conditions, finding that 70% of smiles in genuine conditions were Duchenne smiles, while 83% of smiles in deliberate conditions were Duchenne smiles. Other studies have also found that Duchenne smiles were frequently found in posed conditions: 56% (Ambadar et al., 2009), 60% (Gosselin et al., 2002a), 67% (Schmidt and Cohn, 2001), and 71% (Gunnery et al., 2013). Moreover, this type of smile also appears when watching negative emotional videos (Ekman et al., 1990) and when failing in a game context (Schneider and Josephs, 1991). Some scholars have argued that AU6 may mainly reflect a higher emotional intensity, but not serve as a means of distinguishing a smile’s spontaneity, because many strong negative expressions also include AU6, such as sadness and pain (Bolzani Dinehart et al., 2005). Krumhuber and Manstead (2009) compared the strengths of Duchenne and non-Duchenne smiles, finding that Duchenne smiles’ intensity rating (from “1” meaning weak to “5” indicating very strong) was 3.11, and non-Duchenne smiles was 0.97; the difference was significant. Such difference was also observed by Gunnery et al. (2013) that Duchenne smiles are typically more intense than non-Duchenne smiles. These findings suggest that Duchenne smiles may only be smiles of a greater intensity, but cannot be equated to spontaneity. Some research pre-defines the Duchenne smile (i.e., a smile with AU6) as genuine and therefore suffers from cycle verification. Thus, we cannot simply rely on AU6 to distinguish between genuine and posed smiles and instead should be cautious when selecting the stimuli used when studying genuine/posed smile recognition.

Moreover, most previous research used static images as stimuli in genuine smile recognition tests, and others used video episodes, taking dynamic information such as duration into consideration. Ekman and Friesen (1982) found that the onset time in false smiles would usually be too short, giving an abrupt appearance to the smile. Weiss et al. (1987) found that participants who were hypnotized to experience pleasure in reaction to a corresponding emotion cue showed smiles with longer and smoother onset actions as compared to when they were simulating pleasure. Hess and Kleck (1990) showed for posed expressions (intentionally employed positive expressions to mask disgust) shorter onset and offset times than for emotion-elicited expressions of felt joy. However, simply considering the onset duration may not help to distinguish whether a smile is genuine (Hess and Kleck, 1994). Krumhuber and Manstead (2009) found that the longer the apex duration of a smile, the more likely it is to be judged as genuine. Other research has suggested that a mouth movement might provide important cues for distinguishing genuine and posed smiles. Guo et al. (2018) found that the movement duration of the lips was very helpful in identifying genuine and posed smiles. Genuine smiles had an obviously longer duration than did posed smiles in terms of onset (1.16 s vs. 0.63 s), apex (2.60 s vs. 1.66 s), and offset (1.23 s vs. 0.79 s) durations, with a total duration of 5.00 s for genuine and 3.09 s for posed smiles. The dynamic nature of the smile, including the mouth movement, served as a useful indicator when distinguishing between posed and genuine smiles. If the dynamic features alone can be a good indicator, it is possible that simply focusing on mouth movement may lead to a good performance.

Another issue is the cognitive mechanisms that operate when determining smile genuineness. Some scholars have suggested that low performance in this area can be explained by perceptual-attentional mechanisms indicating that perceivers are unable to perceptually detect the cues responsible for genuine smile recognition (Gosselin et al., 2002b; Boraston et al., 2008) or simply do not allocate attention to these cues (Perron and Roy-Charland, 2013). Perceptual-attentional mechanisms are a reasonable explanation for poor performance. After all, the cues for distinguishing genuine and posed facial expressions are sometimes very subtle (Ekman et al., 1981, 1988; Krumhuber and Manstead, 2009). Only those talented in detecting subtle cues can perceive them, called “true wizards” by Ekman (Granhag and Strömwall, 2004), though the term “true wizard” was criticized by Bond (2008). Williams et al. (2001) investigated the cognitive strategies operating during smile genuineness recognition, focusing on eye fixation. These researchers found that participants paid more attention (i.e., at a higher frequency and for a longer duration) to AU6 when judging facial expressions (i.e., happiness, sadness, and neutral) as happy. Boraston et al. (2008) found that adults with autism paid less attention (i.e., at a lower frequency and shorter duration) to the eye region, suggesting that it was a lack of attention to AU6 that contributed to misjudgment with regard to smile genuineness. These studies underscore the importance of allocating attention to AU6 when seeking to improve smile recognition. When considering the cultural factors, things become more complex. According to previous research, Chinese and Japanese evaluate the role of the mouth and eyes differently from Westerners. Individuals in collectivistic Eastern society heavily rely on information from the eyes to identify and interpret the meaning of smiles (Liu et al., 2010). One study found that when asking Chinese speakers to judge the Duchenne and non-Duchenne smiles as either real or fake, those who voluntarily stated the eyes to be the most useful source of information are more accurate (71.11 ± 12.31%) than those who preferred the mouth (62.89 ± 11.34%), *p* < 0.05. More interestingly, the accuracy of participants preferring the eyes is negatively correlated with individualism scores but positively correlated with collectivism scores, indicating that individuals in a collectivist society heavily rely on information from the eyes to identify and interpret others’ facial expressions and social intentions (Mai et al., 2011). Based on these studies, it seems that paying attention to and perceptually recognizing the responsible cue is the key in genuine smile recognition. The mouth movement, which has better recognizable feature (clearer contour) than the eye regions, may be a more reliable indicator.

Previous research has shown that people perform poorly with regard to recognizing genuine smiles and that the Duchenne marker is not always a useful cue. Rather, dynamic features might be better for distinguishing between genuine and posed smiles. The lips, with their clear morphological features, are easily recognized in dynamic mode and thus may be a good indicator for recognizing smile genuineness. We would like to test the perceptual-attentional hypothesis by training (instructing) the participants to pay more attention to either the eye region or the mouth movement. Even though observers paid the same amount of attention to a certain region, they have different perceptual difficulties to detect the responsible indicators because mouth movement is more salient than contraction of eye regions. Regarding the stimuli used in the present study, the genuine smiles were genuine in nature (i.e., accompanied by the emotion of happiness or amusement), rather than selected by whether there was an AU6 (as some previous research has done). Therefore, we hypothesized that training people to pay attention to mouth movement and Duchenne mark would enhance their performance in genuine smile recognition, but the mouth movement condition should be even better.

## Methods

### Participants

A power analysis with G^∗^Power 3.1.9.2^1^. indicated *N* = 62 to detect an effect size 0.25 with repeated-measures ANOVA and within-between interaction, with a probability of 1–β = 0.9, α = 0.05. Assuming the possible invalid data or missing data in experiments, we recruited 68 participants ranging in age from 18 to 27 years (*M* = 19.78) took part individually in the experiment. All were students from Wenzhou University and were compensated for their participation. All participants were right-handed.

### Stimuli

**FIGURE 1:**
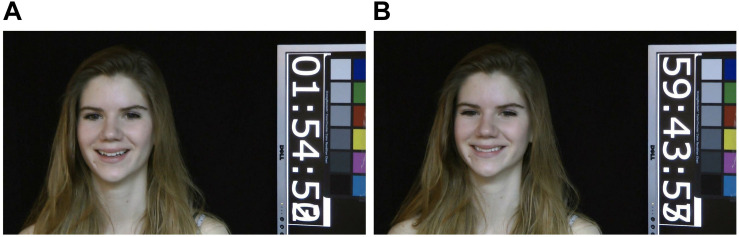
Examples from the UvA-NEMO Smile Database (Dibeklioğlu et al., 2012) which was marked as publishable (but the permission is required to use the images), where **(A)** is a genuine smile and **(B)** a posed smile, illustrating the difficulty in distinguishing genuine smiles from static images.

We selected videos of genuine and posed smiles from the UvA-NEMO Smile Database (Dibeklioğlu et al., 2012) as stimuli in our experiment. The genuine smiles in this database are dynamic video episodes elicited by emotions of happiness or amusement. The database consists of 1,240 smile videos (597 genuine and 643 posed) obtained from 400 subjects (185 female and 215 male), making it the largest smile database in the literature to date. The ages of the subjects varied from 8 to 76 years, with 149 subjects being younger than 18 (in total, offering 235 genuine and 240 posed smiles). The videos were in RGB color and recorded at a resolution of 1,920 × 1,080 pixels, at a rate of 50 frames per second, and under controlled illumination conditions (see examples in Figure 1). For the posed smiles, each subject was asked to pose as realistic an enjoyment smile as possible, after being shown a sample video of a prototypical smile. These genuine smiles of enjoyment were elicited by a set of short, funny video segments shown to each subject for approximately 5 min. The segments all began and ended with neutral or near-neutral expressions. In the experiment, we selected four samples for a practice session and another 80 samples (40 genuine and 40 posed smiles) for the formal session. The distribution of stimulus targets was as follows. The selected stimuli consisted of 40 genuine and 40 posed smiles. There were 42 smiles from males and 38 from females. The minimum age was 8 and the maximum age was 73. The eighteen smiles from children accounted for 22.5% of the total. Another six were from teenagers, or 7.5%, 53 were from adults, or 66.25%, and three were from the aged, or 3.75%.

### Procedure

We used computers with 21-inch LCD monitors (resolution 1024 × 768 pixels) and employed the software package E-Prime 2.0 for stimulus presentation and data collection. The experiment was a 2 (instruction condition: Duchenne marker vs. mouth movement) × 2 (training session: pre-training vs. post-training) mixed design. We randomly selected two genuine and two deliberate smiles from the database for a practice session. We then randomly selected another 40 smiles (20 deliberate and 20 genuine) for the pre-training session and another 40 smiles for the post-training session. We also selected four genuine and four deliberate smiles for instruction (i.e., training) between the pre- and post-training sessions.

A participant was randomly assigned to the condition of either a Duchenne marker or a mouth movement. They were seated in front of a monitor and given instructions regarding the experiment. First, they input their gender, age, and left- or right-handedness and then were required to judge the genuineness of the smiles using only their instinct and experience. The experiment began with four practice trials. They then proceeded to the formal experiment, which consisted of pre- and post-training blocks with 40 trials in each. No feedback was given in practice session or formal session. The stimulus presentations occurred in random order. After the participants finished Block 1 (i.e., the pre-training session), they were given instructions (i.e., training) on how to improve their performance in distinguishing between genuine and deliberate smiles. In the Duchenne marker condition, participants were trained to pay attention to the eye regions. The introduction went like this: *previous research has shown that genuine smiles are accompanied by contraction of the muscles around the eyes and sometimes forms crow’s feet around the eyes*. In the mouth movement condition, participants were trained to focus on the duration and temporal features of the lip corners. The introduction went like this: *Previous research has shown that genuine smiles have longer onset and offset durations with regard to the lips, and smiling lips hold for a longer duration. Posed smiles have shorter onset, apex, and offset durations. Following these conclusions, please distinguish the genuineness of each smile.* After instruction (i.e., training), participants moved to Block 2 (i.e., the post-training session).

For each trial, the stimulus (either a genuine or a posed smile) appeared for several seconds (depending on the duration of the video). After the video played to the end, the participant rated the genuineness of the smile by dragging the mouse on a visual analog scale from -3 (extremely posed) to 3 (extremely genuine). This manipulation transformed the judgment from classification to scale, which provided more information about the participants’ judgments. They chose not only positive or negative but also the intensity of the genuineness. The value is supposed to reflect how confident the participant is when rating the smile as posed or genuine (see Supplementary Material). After the rating, the participant proceeded to the next stimulus presentation.

### Data Analysis

We removed trials with ratings equal to 0 because participants were unable to judge and there was no accuracy. The trials with RT (reaction time) less than 200 ms were also removed because previous research has found that the basic RT is generally no less than 200 ms. The removed data were less than 5% of the total. With these data, we analyzed accuracy (ACC), RT, and scale value using SPSS. We reported partial eta squared as the effect size of the ANOVA.

## Results

**FIGURE 2:**
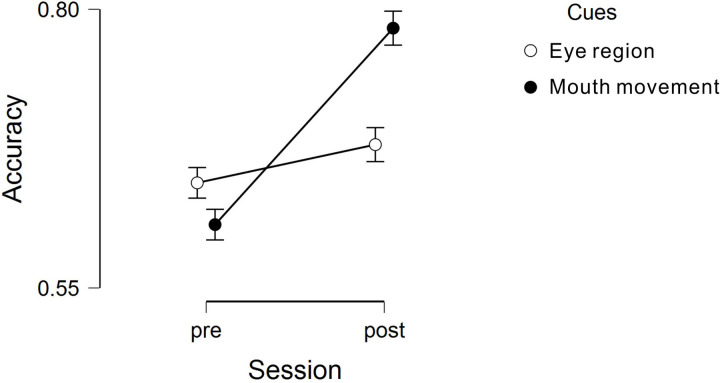
Interaction between factor cue and session (1: pre-training; 2: post-training). The error bar indicates the 95% confidence interval.

A 2 (pre-training/post-training) × 2 (Duchenne marker/mouth movement) repeated-measures analysis of variance (ANOVA) was conducted. First, we considered the accuracy of the judgment. A main effect for training was found, *F*(1, 66) = 5.360, *p* = 0.024, $\eta_{p}^{2}$ = 0.056, indicating that the performance was better post-training (*M* = 0.695, *SD* = 0.140) than pre-training (*M* = 0.661, *SD* = 0.110). A main effect also emerged for the instruction condition, *F*(1, 66) = 23.047, *p* < 0.001, $\eta_{p}^{2}$ = 0.259, showing that the performance was better for the mouth movement condition. The interaction effect (see Figure 2) between training and cue was significant, *F*(1, 66) = 24.062, *p* < 0.0001, $\eta_{p}^{2}$ = 0.267, indicating that training had different effects on different instruction conditions. A simple-effects analysis showed that there was no difference between the Duchenne marker and mouth movement groups in terms of accuracy before training, *F*(1, 66) = 1.222, *p* = 0.273, indicating that the participants from the two groups were randomly assigned and had no differences in terms of ability of distinguishing between genuine and posed smiles. After training, performance for the mouth movement condition improved remarkably (*M* = 0.783, *SD* = 0.94), much better than for the Duchenne marker condition (*M* = 0.606, *SD* = 0.121), *F*(1, 66) = 45.339, *p* < 0.0001. Performance in response to the mouth movement condition was significantly better after training, *F*(1, 66) = 25.323, *p* < 0.0001, while there was no obvious difference (but with the marginal significance) with regard to the Duchenne marker condition after training, *F*(1, 66) = 3.456, *p* = 0.067, showing that training brought remarkable improvement only when the participants were asked to pay attention to the dynamic features of the lips. The results only partly confirmed the hypothesis that “training people to pay attention to mouth movement and Duchenne mark would both enhance their performance in genuine smile recognition, but the mouth movement condition should be even better,” because we found *mouth movement instruction* but not *Duchenne mark instruction* largely enhance their performance.

In addition to the accuracy, we also analyzed the RTs and the scale values. The RT and scale value may reflect how confident the participant is when rating the smile as posed or genuine (see Supplementary Materials).

### Additional Results

However, there may be alternative explanation on “no effect from training for Duchenne markers.” In previous research, many participants pose Duchenne smiles and conversely not all genuine smiles show this marker. If the posed smiles have more AU6 than the genuine ones, it would present an unfair advantage to participants in the Duchenne mark condition. To test this hypothesis, we coded the AU composition and intensity of the AUs according to FACS. The coders rated the intensity of each AU from 0 to 5, where 0 indicated no AU and 1–5 meant intensities A to E, according to FACS. To be more conservative, we classified those level A or weaker as “no AU6” and everything else as “including AU6.” The reliability was calculated by the ICC to be 0.798 for AU6 and 0.576 for AU12. We found that the proportion of AU6 was 92.5% (37 out of 40) for genuine smiles and 17.5% (7 out of 40) for posed smiles. This finding nullifies the alternative explanation that paying attention to the eye region decreases accuracy because there were more AU6 examples in the stimuli for the posed condition.

Before we jump to a conclusion that the participants rely solely on duration, there is still an alternative explanation. In previous research, Thibault et al. (2015) found that mainland Chinese immigrants to Canada did not use the Duchenne marker but rather relied on intensity to judge the genuineness of smiles from members of their own group. Therefore, it is possible that the participants in this study rely on the intensity of AU12 instead of duration. Therefore, we analyzed the intensity of the AUs; the mean was 4.13 for the genuine condition and 3.93 for the posed condition. The difference between the two was insignificant, *t*(78) = 1.194, *p* = 0.236. This finding nullifies the alternative explanation that the intensity of AU12 mainly contributed to recognizing genuine smiles.

## Discussion

The results show that paying attention to mouth movements can help improve performance with regard to distinguishing between genuine and posed smiles. Training to recognize mouth movement had a much larger effect; there was no effect from training for Duchenne markers. This finding contrasts with previous research arguing that the Duchenne marker is the gold standard for genuine smiles, where only those smiles pulling up the lip corners but without Duchenne markers are taken as posed.

Considering the alternative explanations that the distribution of the genuine and posed smiles may affect the results, and the intensity of the smile can be a potential responsible cue, we further analyzed the data and found that the results for Exp. 1 are mainly explained by the duration of lip movement, instead of a biased distribution of AU6 or the intensity of AU12 in posed smiles.

With additional analysis of the stimuli, we found that AU6 alone was actually a strong indicator for genuine smiles. The participants, however, were unable to take this cue into full consideration. Therefore, focusing on AU6 showed no effect not because of a lack of attention but rather because the participants seemed perceptually unable to detect AU6 and use it to help them recognize genuine smiles. Therefore, the present study proposed that the perceptual-attentional hypothesis can explain smile genuineness recognition.

However, we should be cautious to generalize this conclusion to people (both perceiver and the perceived) from other cultures. There might be interaction effects in face perception when considering the perceivers and the perceived faces from different cultures (Matsumoto and Kudoh, 1993; Krys et al., 2016). Based on the present study, we can only say that when Chinese young people try to discriminate the genuine and fake smiles from the Western people’s faces, instructing to pay attention to the mouth movement would benefit. It can also be hypothesized that this effect should have some degree of universality if it works on Chinese young people; after all, we are human beings and share many similarities. Without empirical evidence, we cannot jump to the conclusion that paying attention to mouth movement corners can help improve people’s ability to distinguish genuine from posed smiles. In addition, we must also emphasize that stimuli from the UvA-NEMO Smile Database do not cover all kinds of happy faces, since the smiles were elicited only by funny videos.

## Data Availability Statement

All datasets generated for this study are included in the article/Supplementary Material.

## Ethics Statement

The studies involving human participants were reviewed and approved by the Institutional Review Board in Wenzhou University. The patients/participants provided their written informed consent to participate in this study. Written informed consent was not required to publish potentially identifiable images as these were accessed from the UvA-NEMO Smile Database.

## Author Contributions

W-JY conceived and designed the experiments. Q-NR and J-YH performed the experiments. Q-NR, JL, and W-JY analyzed the data and wrote and revised the manuscript.

## Conflict of Interest

The authors declare that the research was conducted in the absence of any commercial or financial relationships that could be construed as a potential conflict of interest.

## Footnotes

1 G^∗^Power is a tool to compute statistical power analyses. G^∗^Power can also be used to compute effect sizes and to display graphically the results of power analyses, http://stats.idre.ucla.edu/other/gpower/.

## References

[B1] Ambadar Z. Cohn J. F. Reed L. I. (2009). All smiles are not created equal: morphology and timing of smiles perceived as amused, polite, and embarrassed/nervous. *J. Nonverbal Behav.* 33 17–34. 10.1007/s10919-008-0059-5 19554208 PMC2701206

[B2] Bachorowski J. A. Owren M. J. (2001). Not all laughs are alike: Voiced but not unvoiced laughter readily elicits positive affect. *Psychol. Sci.* 12, 252–257. 10.1111/1467-9280.00346 11437310

[B3] Bolzani Dinehart L. H. Messinger D. S. Acosta S. I. Cassel T. Ambadar Z. Cohn J. (2005). Adult perceptions of positive and negative infant emotional expressions. *Infancy* 8 279–303. 10.1207/s15327078in0803_5

[B4] Bond C. F. (2008). Commentary a few can catch a liar, sometimes: comments on Ekman and O’Sullivan (1991), as well as Ekman. O’Sullivan, and Frank (1999). *Appl. Cogn. Psychol.* 22 1298–1300. 10.1002/acp.1475

[B5] Boraston Z. L. Corden B. Miles L. K. Skuse D. H. Blakemore S.-J. (2008). Brief report: perception of spontaneous and posed smiles by individuals with autism. *J. Autism Dev. Disord.* 38 574–580. 10.1007/s10803-007-0421-1 17968644 PMC7615265

[B6] Centorrino S. Djemai E. Hopfensitz A. Milinski M. Seabright P. (2015). Honest signaling in trust interactions: Smiles rated as genuine induce trust and signal higher earning opportunities. *Evol. Hum. Behav.* 36, 8–16. 10.1016/j.evolhumbehav.2014.08.001

[B7] Dibeklioğlu H. Salah A. A. Gevers T. (2012). “Are you really smiling at me? Spontaneous versus posed enjoyment smiles,” in *Proceedings of the European Conference on Computer Vision*, Berlin: Springer, 525–538. 10.1007/978-3-642-33712-3_38

[B8] Ekman P. (2003). Darwin, deception, and facial expression. *Ann. N. Y. Acad. Sci.* 1000 205–221. 10.1196/annals.1280.010 14766633

[B9] Ekman P. Davidson R. J. Friesen W. V. (1990). The duchenne smile: emotional expression and brain physiology: II. *J. Person. Soc. Psychol.* 58 342–352. 2319446

[B10] Ekman P. Friesen W. Hager J. (2002). *Facial Action Coding System (The Manual on CD Rom).* Salt Lake City: Network Information Research Corporation.

[B11] Ekman P. Friesen W. V. (1982). Felt, false, and miserable smiles. *J. Nonverbal Behav.* 6 238–252. 10.1007/bf00987191

[B12] Ekman P. Friesen W. V. O’Sullivan M. (1988). Smiles when lying. *J. Personal. Soc. Psychol.* 54 414–420. 10.1037/0022-3514.54.3.414 3361418

[B13] Ekman P. Hager J. C. Friesen W. V. (1981). The symmetry of emotional and deliberate facial actions. *Psychophysiology* 18 101–106. 10.1111/j.1469-8986.1981.tb02919.x 7220762

[B14] Frank M. G. Ekman P. (1993). *Not all Smiles are Created Equal: The Differences Between Enjoyment and Nonenjoyment Smiles.* Humor: International Journal of Humor Research.

[B15] Frank M. G. Ekman P. Friesen W. V. (1993). Behavioral markers and recognizability of the smile of enjoyment. *J. Personal. Soc., Psychol.* 64 83–93. 10.1037/0022-3514.64.1.83 8421253

[B16] Gosselin P. Beaupré M. Boissonneault A. (2002a). Perception of spontaneous and masking smiles in children and adults: sensitivity to traces of anger. *J. Genet. Psychol.* 163 58–71. 10.1080/00221320209597968 11952265

[B17] Gosselin P. Perron M. Legault M. Campanella P. (2002b). Children’s and adults’ knowledge of the distinction between enjoyment and nonenjoyment smiles. *J. Nonverbal Behav.* 26 83–108.

[B18] Granhag P. A. Strömwall L. A. (2004). *The Detection of Deception in Forensic Contexts.* Cambridge, MA: Cambridge University Press.

[B19] Gunnery S. D. Hall J. A. Ruben M. A. (2013). The deliberate Duchenne smile: individual differences in expressive control. *J. Nonverbal Behav.* 37 29–41. 10.1007/s10919-012-0139-4

[B20] Gunnery S. D. Ruben M. A. (2016). Perceptions of Duchenne and non-Duchenne smiles: a meta-analysis. *Cogn. Emot.* 30, 501–515. 10.1080/02699931.2015.1018817 25787714

[B21] Guo H. Zhang X. H. Liang J. Yan W. J. (2018). The dynamic features of lip corners in spontaneous and posed smiles. *Front. Psychol.* 9:202. 10.3389/fpsyg.2018.00202 29515508 PMC5826373

[B22] Hess U. Beaupré M. G. Cheun N. (2002). “Who to whom and why–cultural differences and similarities in the function of smiles,” in *Mellen Studies in Psychology*, Vol. 4, *An Empirical Reflection on the Smile*, ed. Abel M. H. (New York, NY: Edwin Mellen Press), 187–216.

[B23] Hess U. Kleck R. E. (1990). Differentiating emotion elicited and deliberate emotional facial expressions. *Eur. J. Soc. Psychol.* 20 369–385. 10.1002/ejsp.2420200502

[B24] Hess U. Kleck R. E. (1994). The cues decoders use in attempting to differentiate emotion-elicited and posed facial expressions. *Eur. J. Soc. Psychol.* 24 367–381. 10.1002/ejsp.2420240306

[B25] Kappas A. Descôteaux J. (2003). “Of butterflies and roaring thunder: nonverbal communication in interaction and regulation of emotion,” in *Nonverbal Behavior in Clinical Settings*, eds Philippot P. Feldman R. S. Coats E. J. (Oxford: Oxford University Press), 45–74. Available online at: 10.1093/med:psych/9780195141092.003.0003

[B26] Krumhuber E. G. Manstead A. S. (2009). Can duchenne smiles be feigned? New evidence on felt and false smiles. *Emotion* 9 807–820. 10.1037/a0017844 20001124

[B27] Krys K. Vauclair C.-M. Capaldi C. A. Lun V. M.-C. Bond M. H. Domínguez-Espinosa A. (2016). Be careful where you smile: culture shapes judgments of intelligence and honesty of smiling individuals. *J. Nonverbal Behav.* 40 101–116. 10.1007/s10919-015-0226-4 27194817 PMC4840223

[B28] Liu C. Ge Y. Luo W.-B. Luo Y.-J. (2010). Show your teeth or not: the role of the mouth and eyes in smiles and its cross-cultural variations. *Behav. Brain Sci.* 33 450–452. 10.1017/s0140525x10001263

[B29] Mehu M. (2011). Smiling and laughter in naturally occurring dyadic interactions: relationship to conversation, body contacts, and displacement activities. *Hum. Ethol. Bull.* 26, 10–28.

[B30] Mai X. Ge Y. Tao L. Tang H. Liu C. Luo Y.-J. (2011). Eyes are windows to the Chinese soul: evidence from the detection of real and fake smiles. *PLoS One* 6:e19903. 10.1371/journal.pone.0019903 21647430 PMC3102058

[B31] Manera V. Del Giudice M. Grandi E. Colle L. (2011). Individual differences in the recognition of enjoyment smiles: no role for perceptual–attentional factors and autistic-like traits. *Fron. Psychol.* 2:143. 10.3389/fpsyg.2011.00143 21779265 PMC3134888

[B32] Martin J. Rychlowska M. Wood A. Niedenthal P. (2017). Smiles as multipurpose social signals. *Trends Cogn. Sci.* 21, 864–877. 10.1016/j.tics.2017.08.007 28964655

[B33] Matsumoto D. Kudoh T. (1993). American-Japanese cultural differences in attributions of personality based on smiles. *J. Nonverbal Behav.* 17 231–243. 10.1007/bf00987239

[B34] Otta E. Abrosio F. F. E. Hoshino R. L. (1996). Reading a smiling face: Messages conveyed by various forms of smiling. *Percept Mot. Skills* 82, 1111–1121. 10.2466/pms.1996.82.3c.1111 8823879

[B35] Palmer M. T. Simmons K. B. (1995). Communicating intentions through nonverbal behaviors conscious and nonconscious encoding of liking. *Hum. Commun. Res.* 22, 128–160. 10.1111/j.1468-2958.1995.tb00364.x

[B36] Perron M. Roy-Charland A. (2013). Analysis of eye movements in the judgment of enjoyment and non-enjoyment smiles. *Front. Psychol.* 4:659. 10.3389/fpsyg.2013.00659 24069013 PMC3781329

[B37] Schmidt K. L. Cohn J. F. (2001). Human facial expressions as adaptations: evolutionary questions in facial expression research. *Am. J. Phys. Anthropol.* 116 3–24. 10.1002/ajpa.20001 11786989 PMC2238342

[B38] Schneider K. Josephs I. (1991). The expressive and communicative functions of preschool children’s smiles in an achievement-situation. *J. Nonverbal Behav.* 15 185–198. 10.1007/bf01672220

[B39] Thibault P. Levesque M. Gosselin P. Hess U. (2015). The duchenne marker is not a universal signal of smile spontaneity – but it can be learned! *Soc. Psychol.* 43 215–221. 10.1027/1864-9335/a000122

[B40] Tomkins S. S. (1962). *Affect imagery consciousness, Volume I: The Positive Affects*, Vol. 1 New York, NY: Springer Publishing Company.

[B41] Weiss F. Blum G. S. Gleberman L. (1987). Anatomically based measurement of facial expressions in simulated versus hypnotically induced affect. *Motivat. Emot.* 11 67–81. 10.1007/bf00992214

[B42] Williams L. M. Senior C. David A. S. Loughland C. M. Gordon E. (2001). In search of the “Duchenne Smile”: evidence from eye movements. *J. Psychophysiol.* 15 122–127.

