# Supplementary material for: Focusing on Mouth Movement to Improve Genuine Smile Recognition
Source: Front Psychol. 2020 Jul 28;11:1126. doi: 10.3389/fpsyg.2020.01126 (PMC7399707; doi:10.3389/fpsyg.2020.01126)
Supplement: Supplementary file 1 [file Table_1.DOCX]

**Supplementary Information**

**The normality of the data distribution.**

Kolmogorov-Smirnov test showed that the some of the data were not in normal distribution (see Supplementary Table 1). “Because parametric tests are not very sensitive to deviations from normality, I recommend that you don't worry about it unless your data appear very, very non-normal to you”([McDonald, 2009](#_ENREF_1)). A recent study also showed that in terms of Type I error the F-test was robust in 100% of the cases studied, independently of the manipulated conditions([Mena et al., 2017](#_ENREF_2)). “As long the assumption of normality is not severely violated, the actual Type I error rates approximate nominal rates for t tests and OVA-tests (cf. Boneau, 1960; Glass et al., 1972; Stevens, 2002). However, in the case of data that are severely platykurtic, power is reduced in t tests and OVA-type tests (cf. Boneau, 1960; Glass et al., 1972; Stevens, 2002)”([Nimon, 2012](#_ENREF_3)). In the present study, we would like to investigate possible combination of variables including interactions, and whether they are significant, then the mixed design ANOVA were employed even though the normality is partly violated.

Specifically, for repeated measures ANOVA, we conducted the Mauchly spherical test which showed that data for ACC, RT and Scale value satisfy the spherical hypothesis, with a value of 1.0, which indicates that the data are suitable for repeated-measures ANOVA.

**Supplementary Table 1. Kolmogorov-Smirnov test for normality.**

|  | Session | Eye region | | | Mouth movement | | |
| --- | --- | --- | --- | --- | --- | --- | --- |
|  |  | statistics | df | Sig. | statistics | df | Sig. |
| ACC | Pre-training | .097 | 35 | .200 | .158 | 33 | .035* |
|  | Post-training | .159 | 35 | .025* | .188 | 33 | .004* |
| RT | Pre-training | .251 | 35 | <.001* | .156 | 33 | .039* |
|  | Post-training | .157 | 35 | .028* | .141 | 33 | .095 |
| Scale value | Pre-training | .095 | 35 | .200 | .122 | 33 | .200 |
|  | Post-training | .088 | 35 | .200 | .094 | 33 | .200 |

^*^indicates the distribution is not normal.

**Supplementary results**

For each trial in the experiment, the participant rated the genuineness of the smile by dragging the mouse on a visual analogue scale from -3 (extremely posed) to 3 (extremely genuine) to indicate their judgement. In addition, we recorded reaction time (RT) of participants’ ratings. The RT and scale value may reflect how confident the participant is when rating the smile as posed or genuine.

Only the correct responses of the data were analyzed for RT. We conducted a repeated measures ANOVA on the RT of judgement. The results demonstrate that the main effect of training was significant, *F* (1,66) = 34.038, *p* < 0.0001, $ƞ_{p}^{2}$ = 0.340, indicating that participants’ judgement took less time after training. The main effect of cue was not significant, *F*(1,66) = 0.221, *p* = 0.639, $ƞ_{p}^{2}$ = 0.003. The interaction effect between training and cue for RT was insignificant, *F*(1,66) = 1.884, *p* = 0.175, $ƞ_{p}^{2}$ = 0.028.

We conducted a repeated measures ANOVA for scale values of judgement. The results show that the main effect of training was significant, *F*(1,66) = 36.481, *p* < 0.0001, $ƞ_{p}^{2}$ = 0.356, indicating that participants were more confident in their judgment after training. The main effect of cue was not significant, *F*(1,66) = 2.529, *p* = 0.112, $ƞ_{p}^{2}$ = 0.037. There was an interactive effect between the training and cue factors, *F*(1,66) = 4.628, *p* = 0.035, $ƞ_{p}^{2}$ = 0.066, indicating that training had different effects on different cue conditions. A simple effects analysis showed that the confidence difference between the Duchenne marker and dynamic lip conditions was not significant in the pre-training session, *F*(1,66) = 0.602, *p* = 0.441, while the difference was significant in the post-training session, *F*(1,66) = 4.237, *p* = 0.043, indicating that after training, confidence in the dynamic lip condition seemed to be higher than in the Duchenne marker condition.

**Supplementary Table 2 Descriptive statistics for ACC, RT, and Scale value in the experiment**

|  |  |  | ACC | | RT | | Scale value | |
| --- | --- | --- | --- | --- | --- | --- | --- | --- |
|  |  | *N* | *M* | *SD* | *M* | *SD* | *M* | *SD* |
| Pre-training | Duchenne marker | 35 | .644 | .114 | 1626.513 | 622.925 | 1.433 | .344 |
|  | Mouth movement | 33 | .679 | .103 | 1603.428 | 622.309 | 1.506 | .431 |
| Post-training | Duchenne marker | 35 | .607 | .120 | 1227.062 | 357.000 | 1.585 | .445 |
|  | Mouth movement | 33 | .783 | .094 | 1356.132 | 393.332 | 1.827 | .522 |

Supplementary Table 2 ANOVA Analysis for ACC, RT, and Scale value in the experiment

|  | ACC | | | | RT | | | Scale value | | |
| --- | --- | --- | --- | --- | --- | --- | --- | --- | --- | --- |
|  | *df* | *F* | *p* | $ƞ_{p}^{2}$ | *F* | *p* | $ƞ_{p}^{2}$ | *F* | *p* | $ƞ_{p}^{2}$ |
| Training | 1 | 5.360 | 0.024 | 0.075 | 34.038 | <0.0001 | 0.340 | 36.481 | <0.0001 | 0.356 |
| Cue | 1 | 23.05 | <0.0001 | 0.256 | 0.221 | 0.639 | 0.003 | 2.529 | 0.117 | 0.037 |
| Training ✻ Cue | 1 | 24.062 | <0.0001 | 0.267 | 1.884 | 0.175 | 0.028 | 4.628 | 0.035 | 0.066 |

**Supplementary discussion**

With this visual analogue scale, more information was collected than simply “Yes or No” choice. Training seemed to improve performance from the aspects of confidence, which may be reflected by the RT and scale value. We found that after training, participants became more confident (with significantly less RT and larger scale value) in their judgements, especially with regards to dynamic lip conditions. This may be because the training decreased participants’ hesitation. One reason for this may be that participants believed that instruction from the experimenter would help their judgement. Another reason could be that lip corners have clear contours that allowed for the instructions to be clearly understood.

In experiments with RT as dependent variables, speed-accuracy trade-off is a general problem. However, in out study, after training to focus on mouth movement, the results showed that no such a trade-off but both the accuracy and reaction time improved. It thus strengthens the conclusion that such training is effective in distinguishing the genuine and posed smiles.

**References**

McDonald, J. H. (2009). *Handbook of biological statistics* (Vol. 2): sparky house publishing Baltimore, MD.

Mena, B., José, M., Alarcón, R., Arnau Gras, J., Bono Cabré, R., & Bendayan, R. (2017). Non-normal data: Is ANOVA still a valid option? *Psicothema, 2017, vol. 29, num. 4, p. 552-557*.

Nimon, K. F. (2012). Statistical assumptions of substantive analyses across the general linear model: a mini-review. *Frontiers in psychology, 3*, 322.
